# Biochemical evaluation of X-linked hypophosphatemia and tumor-induced osteomalacia: insights into diagnosis and management

**DOI:** 10.3389/fendo.2025.1702656

**Published:** 2025-11-25

**Authors:** Jorge Díaz-Garzón Marco, Pilar Aguado Acín, Esteban Jodar Gimeno, Pilar Fernández Calle, Vanessa Lopes Martín, María Luisa González-Casaus

**Affiliations:** 1 Department of Laboratory Medicine, University Hospital La Paz, Madrid, Spain; 2 Department of Rheumatology, University Hospital La Paz, Madrid, Spain; 3 Department of Endocrinology and Nutrition, University Hospital Quirón Salud Madrid, Madrid, Spain; 4 Department of Medicine, Faculty of Biomedical and Health Sciences, European University of Madrid, Madrid, Spain; 5 Department of Nephrology, University Hospital Ramón y Cajal, Madrid, Spain

**Keywords:** FGF23, hypophosphatemia, tumor-induced osteomalacia, X-linked hypophosphatemia, XLH

## Abstract

**Introduction:**

X-linked hypophosphatemia (XLH) and tumor-induced osteomalacia (TIO) are characterized by alterations in phosphate metabolism due to elevated levels of fibroblast growth factor 23 (FGF23). These conditions cause significant morbidity due to chronic hypophosphatemia and resulting musculoskeletal disorders.

**Objective:**

This study aims to provide clinical strategies for supporting the diagnosis and management of the biochemical profile of patients with XLH and TIO, addressing key considerations beyond the hypophosphatemia and hyperphosphaturia commonly observed in these conditions and addressing the variability and limitations of current biochemical marker detection methods.

**Materials and methods:**

A literature search focused on studies published in the last ten years. A multidisciplinary team analyzed the data to integrate the findings into clinical best practices.

**Results and discussion:**

The proposed approach emphasizes correctly performing and interpreting tests for serum phosphate, phosphaturia, FGF23, alkaline phosphatase (ALP), parathyroid hormone (PTH), vitamin D, serum calcium, and the calcium-corrected excretion rate. More standardization in screening methods is needed, which affects diagnostic accuracy and management. The recommendations include detailed protocols for patient preparation, sample collection, and interpretation of results.

**Conclusions:**

The recommendations for performing biochemical screening for XLH and TIO promote better clinical practices in patient diagnosis and management. Future research should focus on validating diagnostic methods in diverse populations and standardizing biochemical tests. Multidisciplinary approach to the diagnosis of these patients through the close collaboration of professionals of laboratory medicine and clinical specialties would be pivotal.

## 1 Introduction

X-linked hypophosphatemia (XLH) and tumor-induced osteomalacia (TIO) are rare disorders characterized by elevated levels of fibroblast growth factor 23 (FGF23), which affects phosphate homeostasis. In XLH, mutations in the *PHEX* gene increase FGF23. This leads to decreased renal phosphate reabsorption and serum 1,25-dihydroxyvitamin D levels, causing chronic hypophosphatemia and musculoskeletal disorders such as rickets, osteomalacia, and fractures (1–4). TIO, on the other hand, is caused predominantly by mesenchymal tumors that secrete excess FGF23, although neoplasms of other histological origins have also been described. This results in a pathophysiological cascade characterized by renal phosphate wasting, impaired vitamin D metabolism, and subsequent clinical manifestations such as osteomalacia, fractures, muscle weakness, and bone pain. Both conditions significantly impact the quality of life of patients because of the skeletal and functional alterations they generate (5, 6).

Early diagnosis of XLH and TIO is essential to optimize clinical outcomes and improve the quality of life of patients. In XLH, early diagnosis allows timely initiation of treatment that prevents the worsening of complications such as skeletal deformities and growth deviations during childhood. Early diagnosis also allows the implementation of personalized care plans that reduce the burden of the disease in adulthood (7). In the case of TIO, early detection ensures the implementation of timely treatment and facilitates tumor resection, which can be curative when complete. In addition, early detection contributes to a reduction in the high morbidity associated with the disease, often resulting from an erroneous diagnosis attributable to the nonspecificity of its clinical manifestations (8).

The diagnosis of XLH and TIO relies on a combination of clinical, biochemical, and radiological findings. In the case of XLH, genetic testing can further support the diagnosis (9, 10). However, the most commonly used diagnostic method is the evaluation of biochemical marker levels, which reveals persistent hypophosphatemia, phosphate loss in the kidney and nonsuppressed levels of FGF23 (11–15). Multiple methods are currently used to detect these biomarkers (13, 15–19).

The interpretation of biochemical markers in XLH and TIO remains challenging owing to methodological heterogeneity, preanalytical factors, and patient-specific variables. Differences in sample handling, the type of biological matrix used (e.g., between serum and plasma), and variability in assay performance across laboratories can all contribute to inconsistencies in results (20, 21). International consensus statements have recognized these barriers (12, 22), emphasizing the need for a coordinated, multidisciplinary approach, particularly in complex or refractory cases, to improve diagnostic accuracy and therapeutic monitoring (22, 23). Furthermore, from laboratory medicine, several tools (reference change value [RCV] and personalized reference intervals [pRIs]) have been developed that help and support clinicians in the accurate interpretation of results and disease monitoring while considering previous patient results. These tools are alternatives to classical population reference intervals (popRIs) and are based on biological variation (BV), a concept that defines the variation around the homeostatic set point from a specific individual (within-subject BV) and the variation between the homeostatic set points of different individuals (between-subject BV).The RCV and RI are highly useful, especially for markers that are tightly regulated by homeostatic mechanisms in an individual compared to their distribution in the general population. In other words, the key factor in determining their suitability is the ratio between the within-subject and between-subject BV. Ratios below 0.6 suggest that the use of RCV or pRI is preferable over popRIs. Conversely, when this ratio exceeds 1.4, interpreting results using popRIs is more appropriate (24). However, limited awareness among clinicians regarding the methodological and interpretative constraints of current biomarkers and their interpretation can result in diagnostic errors or suboptimal clinical decisions. A comprehensive interpretation requires the integration of multiple biochemical parameters, such as markers of bone turnover and hormonal regulation, to achieve a more accurate clinical assessment. Given these limitations, laboratory medicine is actively working to improve result interpretation through the development and implementation of formulas that incorporate RCV and pRIs as part of routine clinical practice.

Therefore, this work aims to address these limitations and provide tools, information, and recommendations for the appropriate use of other biomarkers beyond serum phosphates, such as phosphaturia, FGF23, alkaline phosphatase (ALP), parathyroid hormone (PTH), vitamin D, serum calcium, and the calcium-corrected excretion rate, to more effectively address the interpretation of results.

## 2 Materials and methods

A literature search was conducted using the following search strategy: “((“X-linked hypophosphatemia” OR XLH) OR (“tumor-induced osteomalacia” OR TIO)) AND (phosphate OR phosphaturia OR “FGF23” OR “alkaline phosphatase” OR “bone-specific alkaline phosphatase” OR PTH OR “25-hydroxyvitamin D” OR “1,25-dihydroxyvitamin D” OR “vitamin D” OR “biochemical markers”) AND variability AND (“detection methods” OR diagnosis)”, adapted to the following databases: Medline/PubMed, Scopus, and EBSCO.” The search strategy was designed to identify studies published in the last ten years that met scientific relevance criteria. Systematic reviews, clinical trials, clinical practice guidelines, real-life studies, and publications addressing variability in biochemical marker detection methods for diagnosing and managing hypophosphatemia were included. The search flow diagram is shown in **Supplementary Figure 1**.

A multidisciplinary team composed of three specialists in laboratory medicine, a nephrologist, an endocrinologist, and a rheumatologist performed the literature selection and analysis process. These experts contributed their experience in critically analyzing the evidence and integrating the results in this guide.

## 3 Results and discussion

The following biomarkers are mainly used in biochemical analysis to diagnose and manage XLH and TIO: serum phosphate, phosphaturia, FGF23, ALP, PTH, vitamin D, serum calcium, and the calcium-corrected excretion rate. Below are the aspects that the clinician should consider when performing the requested tests correctly and adequately interpreting the values of each of the biomarkers.

**Figure 1:**
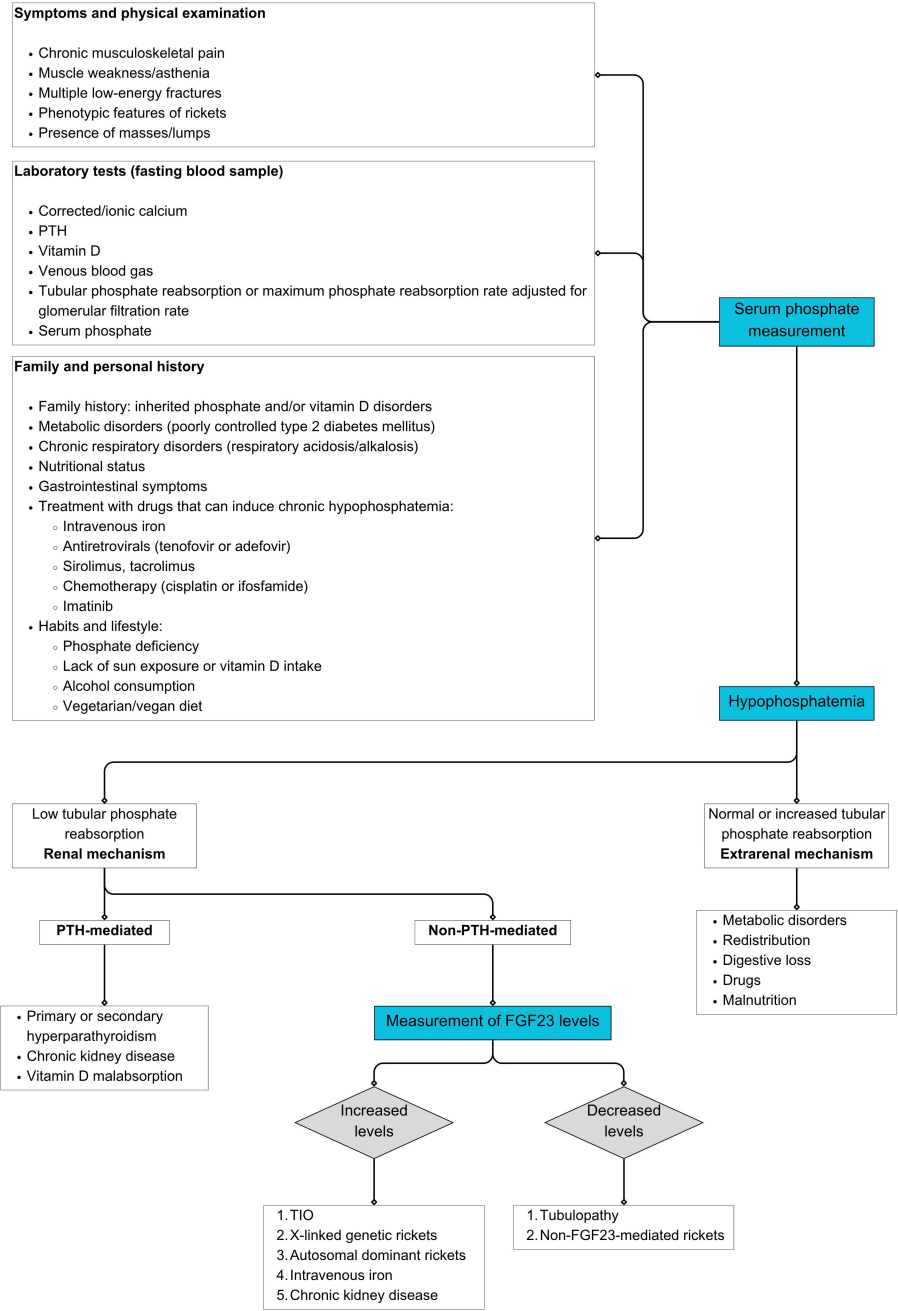
XLH and TIO diagnostic decision algorithms. Prepared by the authors. FGF23: fibroblast growth factor 23; PTH: parathyroid hormone; TIO: tumor-induced osteomalacia; XLH: X-linked hypophosphatemia.

**Figure 2:**
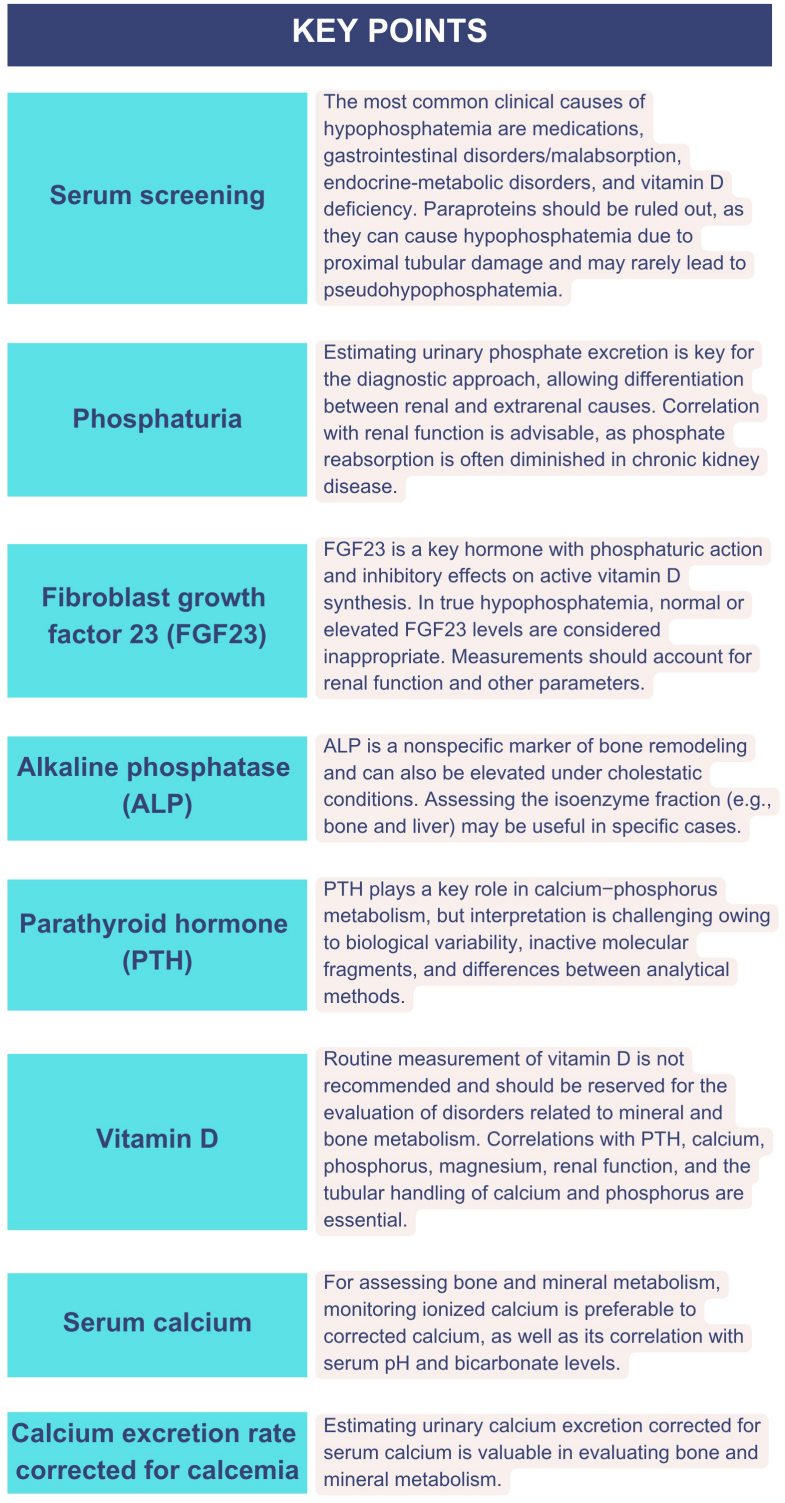
Key biochemical parameters for the evaluation of hypophosphatemia: clinical and analytical considerations.

**Figure 1** details a clinical decision algorithm based on biochemical examination for the diagnosis and management of XLH and TIO. **Figure 2** summarizes the main biochemical parameters involved, highlighting their clinical applications, analytical limitations, and practical interpretation recommendations.

### 3.1 Serum phosphate

#### 3.1.1 Patient preparation

The recommended fasting period for patients is at least 8 h. The intake of food, especially carbohydrates and oral phosphate supplements, may influence the results of serum phosphate measurements (25). A 12-h fast is recommended for more stable and reliable measurements (26).

The patient should report all medications and supplements, especially phosphate supplements, vitamin D, corticosteroids, diuretics, and anticonvulsants, as they may influence phosphate levels (22).

Hemolysis should be avoided during blood collection, as intracellular phosphates may be released, falsely increasing serum levels (27).

#### 3.1.2 Sampling time and matrix type

Serum phosphate levels follow a circadian rhythm, peaking between 2:00 and 5:00 a.m. and reaching their lowest point between 8:00 and 10:00 a.m. It is recommended that the sample be obtained in the early morning hours (26).

A venous blood sample is required, and anticoagulants that chelate calcium or contain phosphates, such as ethylenediaminetetraacetic acid (EDTA) or citrate, must not be used, as they may interfere with the measurement. Serum or plasma should be separated from cells as soon as possible, preferably within 2 h of collection, to avoid the release of intracellular phosphate (28).

#### 3.1.3 Measurement methods

Serum phosphate is measured by colorimetric methods involving the formation of a phosphomolybdate complex. The most commonly used method is the acid–molybdovanadate method, which is relatively well standardized among laboratories, facilitating interchangeability of the results (28).

#### 3.1.4 Reference interval and result interpretation

The common reference interval based on population results (popRI) in adults is 2.5–4.5 mg/dL (0.81–1.45 mmol/L) (**Table 1**). These values may vary slightly depending on the laboratory, the reference population and the method used (29). In both XLH and TIO, chronic hypophosphatemia (levels below 2.5 mg/dL in adults) is present. Notably, phosphate concentrations are higher in children and adolescents than in adults because of bone growth. When interpreting results in pediatric and adolescent populations, age-adjusted reference intervals must be carefully considered to ensure accurate assessment (30). However, hypophosphatemia may also result from various other conditions, including primary hyperparathyroidism, vitamin D deficiency, refeeding syndrome, and renal tubular disorders, since phosphate loss through the kidneys typically occurs in the setting of tubular dysfunction rather than glomerular failure. In contrast, chronic kidney disease usually leads to phosphate retention. Additional factors influencing serum phosphate concentrations include hormonal regulation (e.g., FGF23 and PTH), acid–base status, age, sex, dietary intake, and certain medications, all of which must be considered in the clinical interpretation (31, 32) (**Tables 1**, **2**).

**Table 1:** Reference intervals.

Biomarker	Adults	Children
Serum phosphate	2.5**–**4.5 mg/dL	Newborns: 4.5**–**6.5 mg/dL 1–5 years: 4.0**–**6.0 mg/dL 6–12 years: 3.5**–**5.5 mg/dL Adolescents (13–18 years): 3.5**–**5.5 mg/dL
Phosphaturia (TmP/GFR)	<20%	<20%
iFGF23	30–100 RU/mL	30–100 RU/mL
PTH	10–65 pg/mL	10–65 pg/mL
ALP	44–147 IU/L	Newborns: 150–420 IU/L 1–10 years: 130–375 IU/L Adolescents: 45–300 IU/L
25(OH)D	20–50 ng/mL	20–50 ng/mL
1,25(OH)_2_D	18–72 pg/mL	20–85 pg/mL
Total calcium	8.6**–**10.3 mg/dL	8.8**–**10.8 mg/dL
Corrected calcium	8.5**–**10.2 mg/dL	8.7**–**10.6 mg/dL
Ionic calcium	4.6**–**5.3 mg/dL	4.4**–**5.3 mg/dL
Calcium excretion rate corrected for calcemia	<1%	<1%

1,25(OH)₂ D, 1,25-dihydroxyvitamin D; 25(OH) D, 25-hydroxyvitamin D; ALP, alkaline phosphatase; cFGF23, c-terminal FGF23; FGF23, fibroblast growth factor 23; iFGF23, intact FGF23; PTH, parathyroid hormone; TmP/GFR, threshold of phosphate reabsorption/glomerular filtration rate. The CALIPER database provides reference standards to assist with the interpretation of laboratory test results in pediatric patients (https://caliperproject.ca/caliper/database/) (33).

**Table 2:** Factors influencing the interpretation of biomarkers (prepared by the authors).

Biomarker	Factors to consider	Details
Serum phosphate	Renal function	Glomerular filtration rate affects phosphate levels. Renal failure may lead to hyperphosphatemia.
	Regulatory hormones	FGF23 increases renal phosphate excretion. PTH increases renal phosphate excretion and may influence serum levels.
	Acid–base status	Alkalosis and acidosis may affect phosphate distribution and levels.
	Physiological variations	Age, sex, and pregnancy may influence phosphate levels.
	Diet and nutrition	Dietary phosphate intake may affect serum levels, although the kidneys usually adjust excretion to maintain homeostasis.
	Medications	Diuretics, herbal products, antacids containing aluminum or magnesium, insulin, and glucose may alter phosphate levels.
Phosphaturia	Renal function	GFR affects phosphate excretion. Renal failure may lead to hyperphosphatemia and alter interpretation.
	Acid–base status	Acidosis and alkalosis may influence renal phosphate excretion.
	Physiological variations	Age, sex, and hormonal status may influence phosphate excretion.
	Diet and nutrition	Dietary phosphate intake may affect serum and urinary levels. The patient should maintain a consistent diet prior to testing.
	Medications	Diuretics, corticosteroids, and other medications may alter phosphate excretion.
FGF23 (iFGF23 and cFGF23)	Sample stability	FGF23 is a labile protein; therefore, sample handling and storage is critical to obtaining accurate results. cFGF23 is more stable than iFGF23.
	Analytical interferences	The presence of heterophile antibodies or rheumatoid factors may interfere with immunoassays.
	Renal function	Renal failure may affect FGF23 levels, as its elimination is decreased. In patients with chronic kidney disease, FGF23 levels may be elevated. Take into account phosphate levels in inappropriately normal or nonsuppressed values of FGF23.
	Physiological variations	Age and hormonal status may influence FGF23 levels. Children and adolescents may have higher levels due to active bone growth.
	Medications	Phosphate and vitamin D therapy may influence FGF23 levels.
	Genetic considerations	Mutations in genes related to FGF23 metabolism (*PHEX, DMP1*, and*ENPP1*) may affect its levels and activity.
PTH	Renal function	Renal impairment may affect PTH levels and their interpretation.
	Physiological variations	Age, sex, and circadian rhythms may cause variations in hormone levels.
	Medications	Some treatments such as anticonvulsants, bisphosphonates, and lithium may influence PTH levels.
	Mineral metabolism	Alterations in calcium and phosphorus metabolism should be considered when evaluating results.
ALP	Age and growth	In children and adolescents, elevated levels may be normal due to bone growth and development.
	Pregnancy	An increase in alkaline phosphatase may occur due to production of the placental isoenzyme.
	Liver function	Liver diseases may also lead to elevated levels of alkaline phosphatase. In such cases, distinguishing between hepatic and bone isoforms is essential to avoid misinterpretation, particularly when evaluating bone turnover.
	Renal function	Renal impairment can influence phosphate metabolism and FGF23 clearance, and should always be considered when interpreting biochemical results.
	Medications	Some medications such as anticonvulsants, antidepressants, and oral contraceptives may affect alkaline phosphatase levels
	Additional bone conditions	Diseases such as Paget’s disease, hyperparathyroidism, bone fractures and bone metastases may elevate alkaline phosphatase.
	Physiological variations	The alkaline phosphatase level may vary with age, sex, and during certain life stages such as puberty.
Vitamin D	Seasonality and latitude	Vitamin D levels may vary by season and geographic latitude due to variation in sunlight exposure.
	Age and skin	Older people and those with darker skin have a lower ability to synthesize vitamin D in the skin.
	Obesity	Excess adipose tissue can decrease circulating levels of vitamin D due to its sequestration in fatty tissue.
	Renal function	Renal impairment may affect vitamin D levels and their interpretation.
	Analytical interferences	Some medications and conditions can interfere with immunoassays, causing falsely elevated or decreased results.
	Diet and nutrition	Dietary intake and intestinal absorption affect vitamin D levels.
	Supplementation and treatment	Regular monitoring is advisable in patients receiving vitamin D supplements to avoid toxicity.
Serum calcium	Albumin levels	Hypoalbuminemia reduces measured total calcium without affecting ionized calcium.
	Blood pH	Variations in pH alter calcium binding to proteins. Alkalosis increases binding, reducing ionized calcium; acidosis has the opposite effect.
	Renal function	Chronic renal failure can cause hypocalcemia due to decreased production of active vitamin D and phosphorus retention.
	Hormonal and metabolic status	Excess FGF23 in XLH and TIO reduces vitamin D activation, which may impact calcium absorption and the serum level.
	Physiological variations	Age, sex, pregnancy, and nutritional status can influence calcium levels.
	Analytical considerations	For ionic calcium, careful sample handling is vital to avoid preanalytical errors that affect the result.
Corrected calcium excretion rate	Renal function	Renal failure can alter calcium excretion and affect the interpretation of the index
	Hydration and urinary volume	Fluid intake influences urine concentration and volume, affecting calcium excretion.
	Diet and sodium consumption	High sodium diets increase urinary calcium excretion. Dietary calcium intake should be considered when interpreting the results.
	Medications	Diuretics (loop and thiazide), corticosteroids, and anticonvulsants can modify calcium excretion.
	Acid–base status	Alterations in the acid–base balance may affect renal calcium excretion.
	Physiological variations	Age, sex and hormonal status can influence calcium excretion. Postmenopausal women may have changes in calcium metabolism.
	Proper sample collection	Errors in 24-h urine collection (loss of samples or incorrect timing) may invalidate results. Use of preservatives in the urine container may be necessary to preserve the sample.

ALP, alkaline phosphatase; cFGF23, c-terminal FGF23; FGF23, fibroblast growth factor 23; GFR, glomerular filtration rate; iFGF23, intact FGF23; PTH, parathyroid hormone; TIO, tumor-induced osteomalacia; XLH, X-linked hypophosphatemia.

In the presence of persistent hypophosphatemia, high urinary phosphate excretion indicates inadequate renal phosphate loss, typical of XLH or TIO, mediated by FGF23. Interpretation always requires contextualizing the phosphaturia result with the serum phosphate level and using indices such as TmP/GFR to quantify the tubular defect (11).

The between- and within-subject BV of serum phosphate were moderate (7.7% and 10.7%, respectively). Given these values, strategies based on both population-based reference intervals (for diagnostic purposes) and reference change values (RCVs, for monitoring individual patients over time) can be appropriate tools for interpreting results, depending on the clinical context (**Table 3**) (34).

**Table 3:** Biological variability of biomarkers (prepared by the authors).

Biomarker	Biological variation
Serum phosphate	Moderate to high
Phosphaturia	High
FGF23 (iFGF23 or cFGF23)	High
PTH	Moderate to high
ALP	Moderate
25(OH)D	High or inappropriately normal
1,25(OH)_2_D	Moderate to high
Serum calcium *	Low to moderate
Corrected calcium excretion rate	Moderate to high

1,25(OH)_2_D, 1,25-dihydroxyvitamin D; 25(OH)D, 25-hydroxyvitamin D; ALP, alkaline phosphatase; cFGF23, c-terminal FGF23; FGF23, fibroblast growth factor 23; iFGF23, intact FGF23; PTH, parathyroid hormone; *, Includes total serum calcium, ionic serum calcium and corrected calcium.

### 3.2 Phosphaturia

#### 3.2.1 Patient preparation

The patient should maintain his or her usual diet for several days before the test, avoiding significant changes in phosphate intake. The patient should avoid phosphate and vitamin D supplements unless they are part of the prescribed treatment and report all the medications and supplements being taken, especially diuretics, phosphate, vitamin D, and corticosteroids, as they may influence phosphate excretion. Maintaining regular fluid intake to ensure adequate diuresis is advisable (35).

#### 3.2.2 Sampling time and urine collection

The recommended method for assessing phosphaturia is the analysis of a fasting second morning urine sample, ideally obtained 2 h after the first void (36, 37). Although the tubular maximum phosphate reabsorption per glomerular filtration rate (TmP/GFR) calculated from 24-h urine collection shows suboptimal agreement with the second void TmP/GFR, the differences are not clinically significant, and 24-h urine collection can be used as an alternative in adult patients with urine phosphate wasting (38). Paired fasting plasma samples and urine samples for phosphate and creatinine measurements are required.

#### 3.2.3 Measurement methods

For serum, urinary phosphate is measured by colorimetric methods (12). The TmP/GFR is the most reliable parameter for assessing renal phosphate transport and phosphate reabsorption efficiency in the kidneys. This parameter is calculated from phosphate and creatinine measurements in paired fasting blood and urine samples. Fractional tubular reabsorption of phosphate (TRP) (39, 40) assesses the amount of phosphate reabsorbed by the kidneys in relation to the glomerular filtration rate (GFR). Tubular reabsorption of phosphate per deciliter of the glomerular filtration rate (TP/GFR) is calculated as the difference between the phosphate rate (PPO4) and the phosphate excretion rate (EIPO4) (41).

The formulas to calculate the TRP and TmP/GFR are as follows:


$$TRP=1-(\frac{U_{p}}{P_{p}}\;\times \frac{P_{cr}}{U_{cr}})$$

$$TmP/GFR\;=P_{p}-(\frac{U_{p}}{U_{cr}}\;\times \;P_{cr})$$

When the TRP is > 85%,


$$TmP/GFR\;=P_{p}-(\frac{0.3\;\times \text{TRP}\;}{1-(0.8\;\times \text{TRP})}\;)\times \;P_{p}$$

where P: plasma; p: phosphate; U: urine; cr: creatinine.

Although less commonly used, fractional phosphate excretion (FEPi) can also be calculated for more precise information, which relates the amount of phosphate excreted from the urine to the amount filtered by the kidneys, compared with phosphaturia (the amount of phosphate excreted in the urine). The calculation method is as follows (42):


$$FEPi\;(\%)=(\frac{Urinary\;Phosphate\;(\frac{mg}{dL})\times \;Serum\;creatinine\;(\frac{mg}{dL})\;}{Serum\;phosphate\;(\frac{mg}{dL})\times \;Urinary\;creatinine\;(\frac{mg}{dL})})\times 100$$

In pediatric and young adult populations, the estimation of GFR has progressed from the Schwartz equation (43) to more precise formulas. Currently, the Chronic Kidney Disease in Children under 25 years (CKiD U25) equations are recommended for this purpose. The CKiD U25 equations enable GFR estimation using either serum creatinine or cystatin C, which are calibrated according to the standards set by the International Federation of Clinical Chemistry (IFCC) (44, 45).

#### 3.2.4 Reference interval and result interpretation

The popRI for TmP/GFR in adults is 2.60–3.80 mg/dL (46), and lower values indicate renal phosphate wasting. An FEPi of 20% or higher indicates decreased tubular phosphate reabsorption (increased phosphaturia) (47) (**Table 1**). These intervals may vary slightly depending on the laboratory, the reference population and the method used (48).

Increased phosphaturia indicates renal phosphate loss. However, because hypophosphatemia can result from various etiologies, identifying the underlying cause in each patient is necessary. The evaluation of phosphaturia is essential for determining whether hypophosphatemia is attributable to renal failure (49) or is due to other factors, such as insufficient phosphate intake (especially in individuals with protein-energy malnutrition) (50). These findings, along with hypophosphatemia and elevated FGF23 concentrations, support the suspected diagnosis of XLH. In the case of TIO, increased phosphaturia is due to excess FGF23 produced by the tumor. Tumor resection usually normalizes concentrations. Other causes that can lead to increased phosphaturia include excess FGF23, hyperparathyroidism, Fanconi syndrome, mutations in genes involved in phosphate transport, renal failure, acid–base imbalances and dietary factors. Age and sex also influence serum phosphate concentrations through physiological mechanisms: phosphate tends to decrease with age and is generally lower in men than in women, which must be considered when interpreting results. These changes do not necessarily indicate a pathological process, but they may affect the clinical threshold for defining hypophosphatemia (42, 51, 52) (**Table 2**).

For result interpretation, whether the BV of phosphaturia is high either within or between subjects (20–30%) (53) must be considered, so reference limits or guideline recommendations on the basis of outcome studies must be considered for its correct interpretation (12) (**Table 3**).

### 3.3 Fibroblast growth factor

#### 3.3.1 Patient preparation

Restricting dietary intake and activity is not strictly necessary. However, some studies suggest that fasting may minimize variations in FGF23 levels. The patient should report all medications and supplements, especially phosphate supplements, vitamin D, and medications that may affect mineral metabolism, as these may influence FGF23 levels (54).

#### 3.3.2 Sampling time and matrix type

Circulating intact FGF23 shows significant diurnal variation, reaching its highest value in the early morning and nadir value in the evening, with a mean decrease of 30%, possibly following the circadian rhythm of bone turnover as a bone-derived protein (21). Therefore, the time of sampling is important to avoid the influence of this variation in the interpretation of their results. Considering that intact FGF23 levels peak in the early morning, the optimal time of sample collection is between 7:00 and 10:00 a.m. (54, 55).

Blood samples obtained by the standard phlebotomy procedure in standard EDTA tubes are required. Avoiding hemolysis during blood collection is essential, as this may interfere with FGF23 measurement (54). Although several studies recommended coating blood collection tubes with protease inhibitors to prevent preanalytical degradation (21), subsequent reports indicated that intact FGF23 does not suffer immediate loss of the FGF23 signal and does not require blood withdrawal in these specific containers (56). Nevertheless, the FGF23 concentration decreases when centrifugation is delayed by up to 8 h, and samples should be processed quickly to separate the plasma. If not analyzed immediately, the plasma should be stored at -70 °C, as FGF23 is sensitive to degradation at relatively high temperatures. Repeated freeze–thaw cycles should be avoided (57).

#### 3.3.3 Measurement methods

After its synthesis, some FGF23 molecules are cleaved inside osteocytes by proteases (furines) to yield N- and C-terminal protein fragments. As a consequence of this balance between bone transcription and proteolysis, FGF23 circulates both intact FGF23 (iFGF23) (responsible for biological activity) and N- and C-terminal peptides (58).

Two types of commercial methods are available for measuring FGF23 in human plasma: “intact FGF23” (iFGF23), which recognizes only the active full-length protein, and “C-terminal FGF23” (cFGF23), which detects both iFGF23 molecules as C-terminal fragments derived from proteolytic cleavage. Although iFGF23 assays reflect biological activity, cFGF23 assays represent bone transcription but not necessarily this activity, as they are influenced by posttranslational processing or degradation of FGF23. Occasionally, FGF23 measurements by both methods could be used to assess possible alterations in these posttranslational or cleavage processes. Unfortunately, the lack of standardization or harmonization between commercial enzyme immunoassays (ELISAs) makes comparisons and transferability between results from different laboratories impossible. ELISAs do not include reference intervals; moreover, the results are expressed in different units. Adding to this problem, these ELISAs are available for research use only (RUO). In 2017, a fully automated FGF23 assay became available for diagnostic use (“*in vitro* diagnostic” IVD). This method is the only automated chemiluminescent assay (CLIA) developed and has been extensively validated (54, 59, 60).

#### 3.3.4 Reference interval and result interpretation

FGF23 values may vary depending on the laboratory and the method used (61). As research use only (RUO) methods (62), commercial ELISAs do not include popRIs, but some authors have assessed a plasma iFGF23 reference range between 10 and 50 pg/mL. The lower and upper reference limits for cFGF23 are 20 and 100 RLU/mL, respectively, because both intact hormones and fragments are measured.

For the CLIA iFGF23 assay validated for clinical use, the popRI is 23.2–95.4 pg/mL, without significant differences according to sex or age (59, 61) (**Table 1**).

In XLH, FGF23 levels are elevated because of loss-of-function mutations in the *PHEX* gene. Although the exact pathogenic mechanism remains unclear, *PHEX* inactivation may lead to increased FGF23 expression, potentially mediated by extracellular matrix proteins such as MEPE. This contrasts with autosomal dominant hypophosphatemic rickets (ADHR), in which FGF23 elevation is attributed primarily to impaired degradation. However, despite being an enzyme, the expression rather than the degradation of FGF23 seems to be affected. In TIO, FGF23-producing mesenchymal tumors secrete excessive amounts of FGF23. Therefore, increased levels of FGF23 may suggest these conditions. However, in some cases, FGF23 values may appear within normal ranges, which should not be interpreted in isolation. In patients with XLH, these values may be inappropriately normal or nonsuppressed, which contrasts with the presence of hypophosphatemia and reflects an alteration in the regulation of calcium–phosphorus metabolism (12). High values may also be associated with hypophosphatemia due to decreased renal tubular reabsorption of phosphate and reduced synthesis of 1,25-dihydroxyvitamin D in renal diseases. Other factors influencing FGF23 concentration include sample stability, analytical interference, the use of certain medications (in which FGF23 is not a reliable marker of treatment efficacy), and genetic mutations. Although higher FGF23 concentrations have been observed in older adults, this association is attributable primarily to an age-related decline in renal function (63) (**Table 2**).

The within-subject BV of FGF23 is moderate (14%), and the between-subject BV is high (22.5%) (64), so the RCV or personalized RI should be preferred for patient monitoring instead of popRI (65) (**Table 3**). Furthermore, although elevated values of FGF23 are observed in TIO and XLH, differences exist between these two diseases. Higher concentrations of FGF23 are usually observed in TIO due to ectopic production by mesenchymal tumors, which rapidly decreases after tumor removal (66).

### 3.4 Alkaline phosphatase and bone alkaline phosphatase

#### 3.4.1 Patient preparation

Fasting is not necessary for ALP or BALP measurement, as food intake does not significantly affect ALP concentrations. The patient should report any medications or supplements, especially anticonvulsants, steroids, or vitamin D and calcium supplements, as these may influence ALP levels. Hemolysis should be avoided during blood collection, as intracellular phosphates may be released and falsely increase serum levels (67).

#### 3.4.2 Sampling time and matrix type

Both ALP and BALP have minimal variation throughout the day, so samples can be obtained at any time. A venous blood sample is required for either serum or lithium heparin-plasma. Samples with EDTA or citrate are not recommended because they may interfere with the enzyme measurement of ALP. The sample should be processed as soon as possible. If not analyzed immediately, the serum may be stored refrigerated at 2–8 °C for several days (67).

#### 3.4.3 Laboratory methods

ALP is measured by colorimetric enzymatic methods that quantify the release of a colored product from a specific substrate, such as p-nitrophenylphosphate. Isoenzyme determination can occasionally be performed to identify specific isoenzymes (bone, liver, and placental) by electrophoresis or immunoassays when the release of ALP could be related to other causes (67). Laboratories that support XLH and TIO diagnosis should have an ALP standardized IFCC method.

For a more accurate assessment in adults, serum bone-specific ALP (BALP) is preferable (68), but its measurement in clinical routine is much less standard, and its availability may be limited in some hospitals. There are several methods to measure BALP, such as heat inactivation, electrophoresis, HPLC, or immunoassays. Its measurement is complex due to a 20% cross-reactivity for liver ALP. Automated immunoassays based on monoclonal antibodies specific to bone are the most suitable method for use in a clinical laboratory. Some of these immunoassays measure the mass of the enzyme (μg/L), whereas some others measure the activity of the BALP enzyme (U/L).

#### 3.4.4 Reference interval and result interpretation

The popRI for ALP in adults is 44–147 IU/L. In children, ALP levels are generally higher due to rapid bone growth (65, 69) (**Table 1**). These values may vary slightly depending on the laboratory and method used (70). The popRI for BALP adults reported with the use of immunoassays that measure the mass of the enzyme is 5,5- 24,6 μg/L. BALP activity results are not interchangeable due to the lack of harmonization.

In both XLH and TIO, patients often have elevated ALP. Elevated ALP generally indicates increased osteoblastic activity or possible liver disease. However, distinguishing between ALP and bone-specific ALP is important. The first is a general measurement of ALP, and although much of this measurement is derived from bone, its contribution varies according to age. In children, approximately 80–90% of ALP is derived from bone due to the high osteoblastic activity associated with growth, whereas in adults, approximately 50% of ALP originates from the liver. This can make interpreting elevated ALP levels difficult, as overlap may exist between ALP derived from bone activity and other sources, such as the liver (71). So that for a more accurate assessment in adults, BALP is preferable (68). Its concentration depends only on its rate of release from the osteoblasts, and it reflects the mineralization phase of bone formation through stimulation of pyrophosphate hydrolysis.;. Therefore, ALP levels need to be interpreted along with other parameters, such as serum phosphate, calcium, PTH, vitamin D, and FGF23 levels, in addition to clinical and radiological findings (72).

In patients with XLH and TIO treated with burosumab, long-term ALP results are useful for assessing clinical evolution and therapeutic efficacy since their decrease reflects an improvement in osteoblastic activity and normalization of bone metabolism (12).

When interpreting values, the ALP BV in serum may vary due to osteoblastic activity, bone growth in children, liver functions, and other physiological conditions; therefore, various factors must be considered in its interpretation (72) (**Table 2**).

The within- and between-subject BV of ALP are low (6.6%) and high (35.6%), respectively. Large differences exist between the set points of individuals, but their homeostatic regulation is narrow; therefore, the RCV or personalized RI must be used for patient monitoring instead of the population RI to detect changes in enzyme concentration (73) (**Table 3**).

### 3.5 Parathyroid hormone

#### 3.5.1 Patient preparation

The patient should fast for 8–12 h before the sample is taken, as food intake can influence PTH levels. The patient should avoid taking biotin supplements at least 8 h prior to sample collection, as they can interfere with immunoassays. Calcium and vitamin D supplementation should be carefully individualized and monitored. In patients receiving active forms of vitamin D (e.g., calcitriol), temporary suspension may be considered under medical supervision in cases of hypercalcemia or hypercalciuria. However, native vitamin D (cholecalciferol) should not be discontinued, as its deficiency can contribute to secondary hyperparathyroidism and worsen the phosphate balance (74).

#### 3.5.2 Sampling time and matrix type

The sample should preferably be obtained in the early morning hours due to the circadian variation in PTH (75).

Blood samples obtained by the standard phlebotomy procedure in standard EDTA or serum gel tubes are used for analysis. Importantly, the PTH concentration is significantly greater when blood samples are collected via central vascular access. PTH in EDTA plasma has greater stability at room temperature than that in serum and can be stored at 4 °C and analyzed within 72 h of venipuncture. The main practical advantage of serum is that calcium and bone-alkaline phosphatase (analytes that cannot be measured in EDTA plasma) can be quantified in the same sample, but samples must be centrifuged, and the serum should be separated as soon as possible. Serum must be analyzed within 3–4 h of venipuncture or stored at -20 °C (76). PTH measurements in samples collected in “rapid serum tubes” (RSTs) are not recommended, as a negative bias (approximately –15%) in PTH results has been reported (77).

#### 3.5.3 Measurement methods

Owing to their advantages, such as full automation, short turnaround time, and high specificity and sensitivity, immunoassays are the most common method for measuring PTH. Nevertheless, they also present diverse problems that could lead to misinterpretation and inadequate clinical decisions (78). Circulating PTH is a mixture of different molecular forms, including intact or whole peptides 1–84 and various truncated forms, such as PTH 7–84 and smaller fragments. Furthermore, the same PTH undergoes posttranslational oxidation and phosphorylation processes between amino acids 8 and 18. These truncated peptides of PTH are recognized to various extents by different immunoassays, leading to important interassay variability, especially in those conditions associated with excessive increases in PTH (such as chronic kidney disease). The second generation of PTH immunoassays, inappropriately called “Intact PTH” assays, measure the active 1–84 PTH and the 7–84 truncated fragments. To avoid such interference, third-generation assays, known as “whole or Bio-intact PTH”, were developed; however, these latter methods also recognize the posttranslational phosphorylated PTH forms (named amino-PTH). The oxidized and nonoxidized forms of PTH cannot be distinguished by current immunoassays (78).

Considering these differences, discrepancies between intact and whole PTH immunoassays are expected, as they measure different PTH forms. Nevertheless, significant inconsistences exist among different intact assay results because they were standardized and calibrated against diverse standards from distinct origins. This situation leads to a lack of transferability in PTH results that are minimized to “whole or Bio-intact PTH immunoassays”, as they were calibrated or referenced to the WHO International Standard PTH 1–84 (NIBSC code: 95/646 (79)). More recently, a method based on liquid chromatography–tandem mass spectrometry (LC–MS/MS) for the quantitative analysis of PTH has been developed as a promising candidate reference procedure method (RPM) (80).

#### 3.5.4 Reference interval and result interpretation

popRI must be determined for each matrix (serum or EDTA plasma) and analytical method. In adults, the most commonly used popRI for PTH is 10–65 pg/mL. When a bio-intact PTH method is used, the popRI of 1–84PTH is 4–40 pg/mL. In children, slight variations should be taken into account depending on age and bone development (65) (**Table 1**).

These values may vary depending on the laboratory and method used due to the lack of standardization and interchangeability among intact PTH assays.

In XLH, PTH concentrations may be normal or slightly elevated. Although increased FGF23 values initially inhibit parathyroid gland activity, the resulting suppression of the renal of 1,25-dihydroxivitamin D synthesis indirectly stimulates PTH secretion to maintain calcium homeostasis. Additionally, conventional therapy with high doses of phosphate supplementation and active vitamin D analogs increases the risk of secondary and tertiary (hypercalcemic) hyperparathyroidism as well as nephocalcinosis (81).

Similarly, in TIO, the overproduction of FGF23 by tumor and the impact of phosphate salts treatment on PTH secretion must be considered, as it affects the regulation of calcium and the synthesis of calcitriol, which are involved in mineral balance (82).

Other conditions that may increase its concentration are secondary hyperparathyroidism, vitamin D deficiency, hypocalcemia and hyperphosphatemia, renal failure, age, sex, mineral metabolism, and certain medications; thus, various factors must be considered in its interpretation (47) (**Table 2**).

The within-subject and between-subject BV of PTH is moderate (14.7%) and high (28.9%), respectively. Consequently, both the RCV or popRI should be considered for patient monitoring (47, 64, 83) (**Table 3**).

### 3.6 Vitamin D (25-OH vitamin D)

#### 3.6.1 Patient preparation

Fasting is not strictly necessary for measuring vitamin D levels. However, some laboratories may prefer that the patient fast to avoid minor interference. The patient should report all the medications and supplements he or she is taking, especially vitamin D and calcium supplements, as they may influence serum concentrations. An exposure restriction prior to testing is not necessary (12).

#### 3.6.2 Sampling time and matrix type

Considering that B-ultraviolet sunlight exposure is the main source of vitamin D, vitamin D shows seasonal variation, with the highest levels of 25-(OH)D occurring during summer and lower levels occurring during winter and spring. However, vitamin D exhibits a low within-subject BV without significant circadian variations, so the sample can be obtained at any time (12).

A venous blood sample collected in dry or gel serum tubes is required for analysis. Heparin and EDTA tubes could be used but are not recommended because anticoagulants may interfere with vitamin D measurement, especially in immunoassays. Vitamin D metabolites can also be measured in other body fluids or in dried blood spots, but these matrices are not yet standardized (84). Serum should be separated from cells as soon as possible and can be stored without significant loss in concentration for several days at 2–8 °C (or indeed at room temperature) or frozen at -20 °C for long-term storage (85). The effects of repeated freeze–thaw cycles are not significant for the 25-(OH)D concentration. Importantly, when vitamin D has been separated from its binding protein, samples should be protected from light, as vitamin D is sensitive to ultraviolet light, and stored at -80 °C.

#### 3.6.3 Measurement methods

25-Hydroxyvitamin D (25-(OH)D) is the main circulating vitamin D metabolite and represents the pool from which 1,25-(OH)2D can be formed by renal or extrarenal 1-alpha-hydroxylation. For this reason, 25-(OH)D is considered the best indicator of vitamin D status in the body. Most 25-(OH)D circulates as 25-(OH)D3 but also as 25-(OH)D2, a very similar form of vitamin D derived from vegetable sources or supplements. Therefore, the measurement of vitamin D status should encompass both isomers (84).

Total 25-(OH)D can be measured using diverse methods, including immunoassays, protein-binding assays, HPLC-UV or liquid chromatography–tandem mass spectrometry (LC–MS/MS). Automated immunoassay platforms are widely used in clinical laboratories because they are fast and easy to operate, but unfortunately, they lack accuracy. The difficulty of removing vitamin D from its binding proteins and differences in cross-reactivity for 25(OH)D2 have led to differences in both specificity and sensitivity among immunoassays (86). For this reason, more laboratories have turned to LC–MS–MS/MS. This method offers greater specificity with the advantage of its ability to independently measure different vitamin D metabolites (22), but this technique is more complex, expensive, requires trained technicians and, as with automated immunoassays, standardization is still lacking. To minimize the variability and bias in 25-(OH)D measurements, a vitamin D standardization program (VDSP) was developed (87).

#### 3.6.4 Reference interval and result interpretation

Circulating 25-(OH)D levels show a seasonal variation of 20–30%, with the highest values occurring in summer and autumn. The interpretation of their results is recommended on the basis of clinical cut-offs from outcome studies rather than by a popRI, as the risk for several clinical conditions varies across the reference interval. Several scientific societies have proposed cutoffs to define the sufficiency, insufficiency and deficiency of vitamin D on the basis of bone health, osteoporosis, fracture risk and general health. The cutoffs recommended by the International Osteoporosis Foundation are <20 ng/mL, 20–30 ng/mL and >30 ng/mL to define deficiency, insufficiency and sufficiency, respectively, but discrepancies exist with respect to other scientific societies (88).

In XLH and TIO, 25-(OH)D levels may vary depending on sun exposure, dietary intake, and supplementation, factors that also affect the general population. However, FGF23 excess in these patients also induces CYP24A1 promoting both 25OHD and 1,25-(OH)2D catabolism (89). So that, this increases 24 hydroxylation to 24,25(OH)2D could contribute to lower circulating 25(OH)D levels. Osteomalacia is associated with low 25-(OH)D levels. Other factors can influence the interpretation of vitamin D values, such as seasonality, age (25-(OH)D tends to decrease in older adults due to reduced cutaneous synthesis, lower sun exposure, and age-related changes in hepatic and renal function), skin, obesity, kidney disease, analytical interferences, diet, supplementation, or treatment, so various factors must be considered in its interpretation (90, 91) (**Table 2**).

The within- and between-BV 25-(OH)D levels are moderate (6.8%) and high (30.1%), indicating that the results vary greatly between individuals, reflecting biological differences in circulating proteins and polymorphisms of vitamin D receptor (VDR) or vitamin D binding protein (VDBP). However, the 25-(OH)D levels are quite stable within individuals; thus, as in previous measurements (PTH and FGF-23), the RCV or personalized popRI must be considered for 25-(OH)D monitoring (i.e., treatment efficacy) (92) (**Table 3**).

### 3.7 1,25-Dihydroxy vitamin D

#### 3.7.1 Patient preparation

As 25-OHD, fasting is not strictly necessary, and patients should report medication and supplement intake.

#### 3.7.2 Sampling time and matrix type

Serum and plasma can be used to measure 1,25-(OH)2D, but serum is the preferred matrix for this purpose. The storage conditions for 1,25-(OH)2D are less studied than for 25-OHD. One study shows lower stability compared to 25-OHD, with significant decrements after three freeze-thaw cycles (93).

#### 3.7.3 Measurement methods

Its measurement is more challenging than that of 25-(OH)D because it circulates at very low concentrations (22). 1,25-(OH)2D can be measured by immunoassays or LC–MS/MS methods. However, there is no reference method as there is for 25-OHD, and standardization is lacking.

#### 3.7.4 Reference interval and result interpretation

In healthy adults, 1,25-(OH)_2_D concentrations range from 18 and 72 pg/mL, whereas in children, these values are higher (94). The 1,25-(OH)2D values may vary slightly depending on the laboratory and method used (95).

Although 1,25-dihydroxyvitamin D (1,25-(OH)2D) is the active form of vitamin D, its measurement does not provide additional value for evaluating vitamin D status because 1-alpha-hydroxilation is controlled by PTH, FGF23, or 1,25(OH)2D itself. Circulating 1,25(OH)2D concentration proceeds from the kidney but not from the extra-renal 1-alpha-hydroxilation (not regulated by PTH or FGF23). Therefore, it reflects only the endocrine calciotropic function of this hormone and its measurement should be limited to specific clinical conditions, such as hypocalcemia or hypercalcemia (96).

In XLH, tumor induced osteomalacia and other rare disorders leading to FGF23 excess and hypophosphatemia, 1,25(OH)2D levels may be decreased or inappropriately normal despite hypophosphatemia. Excess FGF23 under these conditions inhibits renal 1-alpha-hydroxylase activity, reducing the conversion of 25-(OH)D to 1,25-(OH)2D in the kidney. At same time, FGF23 promotes 24-hydroxilation of vitamin D leading to an increase in its degradation. Deficiency lead to osteomalacia and adversely affect bone health. Low 1,25-(OH)2D circulating levels are also present in other genetic disorders with loss of function of CYP27B, leading to vitamin D dependent rickets (97).

Conversely, 1,25-(OH)2D levels may be elevated in hyperparathyroidism, sarcoidosis, and some lymphomas, and decreased in chronic renal failure, hypoparathyroidism, and hereditary resistance to vitamin D.

There are no studies regarding the biological variation of 1,25(OH)2D.

### 3.8 Serum calcium

#### 3.8.1 Patient preparation

Patients are recommended to fast for at least 4–6 hours, as food and drink intake can alter blood pH and affect ionized calcium levels. Avoiding hemolysis during blood collection is essential because hemolysis can release intracellular calcium and alter the results (90).

#### 3.8.2 Sampling time and matrix type

For total and corrected calcium, the circadian variations are minimal so that the sample can be obtained at any time of day. In the case of ionized calcium, although diurnal variations are minor, obtaining a sample in the early morning is preferable (98).

A venous blood sample is required for analysis. For total and corrected calcium, a tube without anticoagulant or with a serum-separating gel is required. Anticoagulants that chelate calcium, such as EDTA or citrate, should not be used. For ionized calcium, an anaerobic heparinized tube or a syringe of calcium-balanced lithium heparin for blood gas analysis is required to avoid exposure to air and changes in pH (99).

For total and corrected calcium, the serum should be separated from the cells as soon as possible and may be stored and refrigerated if not analyzed immediately. The sample should be kept anaerobically for ionized calcium and analyzed as soon as possible (ideally within 30 min) to avoid changes in pH and ionized calcium levels (90). Importantly, ionized calcium concentrations can fluctuate with changes in pH, given that the affinity of calcium for protein binding is pH dependent. This adds an additional layer of complexity when interpreting results, especially in pathologies where the pH can be altered (i.e., renal impairment).

#### 3.8.3 Measurement methods

For the determination of total calcium, spectrophotometric colorimetric methods such as orthocresolphthalein complex one (OCPC), in which calcium reacts with OCPC, forming a colored complex that is measured spectrophotometrically, and arsenazo III, which similarly forms a colored complex with calcium, are used. The corrected calcium concentration is calculated using a mathematical formula that adjusts total calcium for serum albumin levels since a portion of calcium is partially bound to proteins. Other authors have used a formula that adjusts the calcium concentration to the total protein concentration. In contrast, ionized calcium is measured directly using ion-selective electrodes (ISEs), which determine the concentration of free (ionized) calcium in the blood using specific electrodes (90).

#### 3.8.4 Reference interval and result interpretation

The total calcium concentration in the population typically ranges from 8.6 to 10.3 mg/dL in adults and 8.8 to 10.8 mg/dL in children, reflecting a global measure of calcium metabolism. Furthermore, ionized calcium, which represents the active form of calcium in the body and is essential for neuromuscular and cardiac functions, is typically between 4.6 and 5.3 mg/dL in adults and between 4.4 and 5.3 mg/dL in children (47, 65) (**Table 1**). These values may vary slightly depending on the laboratory and method used (100).

Corrected calcium provides a more accurate estimate of physiologically relevant calcium in patients with abnormal albumin levels, although direct measurement of ionized calcium is the gold standard for assessing bioactive calcium status (54).

In XLH, total and corrected calcium are usually normal or slightly decreased. Chronic hypophosphatemia may lead to alterations in bone metabolism, but serum calcium is usually maintained within normal values by compensatory mechanisms. Ionized calcium usually remains normal, indicating that physiologically active calcium is within adequate limits. In TIO, total and corrected calcium are usually normal. In advanced cases, severe osteomalacia may lead to hypocalcemia. Ionized calcium may be decreased if significant hypocalcemia is present (47, 65).

Factors that may affect calcium concentrations include albumin concentrations, blood pH, kidney disease, hyperparathyroidism, hypoparathyroidism, vitamin D deficiency, some hormones (such as FGF23), age, sex, and errors in sample processing; therefore, various factors must be considered in the interpretation of results (54) (**Table 2**).

The within- and between-subject BV of total calcium are very low (1.8 and 2.2%) because their homeostatic regulation is essential for life. Additionally, ionized calcium shows a very low BV (1.8 and 2.0%) (101). Although the ratio (0.8) allows the use of either the popRI or pRIs for result interpretation, even small variations in its concentration may have a significant impact on basic physiological functions, highlighting the importance of accurate monitoring and interpretation. Given its tight homeostatic regulation and clinical relevance, the use of RCV or pRI may offer better clinical insight than the popRI does (102) (**Table 3**).

### 3.9 Calcium excretion rate corrected for calcemia

#### 3.9.1 Patient preparation

The patient should maintain their diet for several days before the test, avoiding restrictions or excesses in calcium and phosphorus intake. Calcium and vitamin D supplements should be avoided unless they are part of the prescribed treatment.

#### 3.9.2 Sampling time and urine collection

For the assessment of urinary calcium excretion, the most accurate and preferable option is to measure calcium in a 24-h urine sample collected in a suitable container. Proper instructions on the collection technique are needed to avoid over- or under-collection. Alternatively, overnight urine samples can be used to estimate calcium excretion (103).

Owing to the arduousness of its collection, fractional excretion of calcium (FECa) in spot urine (preferably obtained from the second morning void) could be used as a screening method or for monitoring; however, 24-h urine collection should be used for confirmation when results from spot samples are inconclusive or in complex clinical scenarios. A venous blood sample collected in a tube without anticoagulant or with a serum separator gel is required to measure serum calcium and creatinine for FECa estimation (90).

#### 3.9.3 Measurement methods

To determine urinary and serum calcium levels, colorimetric methods such as OCPC or arsenazo III are used. These methods quantify the total calcium present in the urine by forming colored complexes that are measured spectrophotometrically (12).

The FECa estimates the percentage of filtered calcium that is excreted in the urine and is especially useful when 24-h urine collection is not available. The FECa can be calculated from spot urine samples using the following formula (104):


$$Fractional\;excretion\;of\;calcium\;(FECa\%)=(\frac{Urine\;calcium\;\times \;serum\;creatinine}{Serum\;calcium\;\times urine\;creatinine\;})$$

#### 3.9.4 Reference interval and result interpretation

The reference interval for 24-h urine calcium is 50–300 mg/day. However, to avoid misinterpretation of the results due to over- or under-collection, urine creatinine measurements and the calcium/creatinine ratio are often used. Ratios above 0.2 suggest hypercalcemia.

In healthy adults, the FECa typically ranges between 1% and 2%. Values less than 1% reflect hypocalciuria, as in familial hypocalciuric hypercalcemia (FHH), due to inappropriately high renal calcium reabsorption. Values above this threshold indicate renal calcium loss, as observed in conditions such as primary hyperparathyroidism and (47) (**Table 1**). These values may vary slightly depending on the laboratory and method used (105).

In XLH, patients may present with hypercalciuria due to treatment with phosphate and vitamin D analogs (e.g., calcitriol), which increase intestinal calcium absorption. An elevated index (>1%) suggests a risk of nephrocalcinosis and renal damage. In these cases, supplemental dosage adjustment or treatment modification may be necessary (12).

In TIO, patients may present with hypocalciuria due to decreased renal calcium reabsorption mediated by excess FGF23. A low index (<1%) reflects alterations in calcium metabolism and may aid in the differential diagnosis (12).

Other factors that should be considered include hydration and urinary volume, diet, liver disease, certain medications, age (in adults over 60 years of age, calcium excretion decreases), sex (men generally excrete more calcium than women), hormonal status, acid–base status, or sample collection; therefore, various factors must be considered in its interpretation (90, 106, 107) (**Table 2**).

The BV of urine calcium is high, and as for urine phosphate, the use of the popRI and guideline-recommended cutoffs on the basis of clinical outcomes must be considered for the correct interpretation of the values. Certain commonly used medications, such as loop diuretics, thiazides, and sodium–glucose cotransporter 2 inhibitors (SGLT2is), can significantly alter urinary calcium excretion (90) (**Table 3**).

### 3.10 Genetic study

In the case of XLH, current guidelines recommend performing genetic testing to confirm mutations in the *PHEX* gene whenever accessible. However, when molecular testing is not available, the diagnosis can still be supported by a consistent pattern of biochemical abnormalities, such as persistent hypophosphatemia, elevated or inappropriately normal FGF23 levels, and low renal phosphate reabsorption, alongside clinical and radiological features of rickets or osteomalacia. In such scenarios, family history may provide additional support, although the overall level of evidence is lower (12). In addition, detecting hereditary mutations allows for early diagnosis, facilitating genetic assessment and counseling for family members, which is crucial for preventive management and monitoring of possible asymptomatic carriers within the family (9, 108, 109). Genetic confirmation is therefore not strictly required for diagnosis but adds value when available.

### 3.11 Pharmacological agents

In addition to genetic and tumor-related causes of chronic hypophosphatemia, clinicians should consider pharmacological agents that can influence phosphate levels through either extrarenal redistribution or renal phosphate wasting. These mechanisms can mimic or exacerbate the biochemical phenotype of XLH and TIO, potentially confounding diagnosis and management (110–112). **Table 4** summarizes the pharmacological agents associated with hypophosphatemia, organized by their mechanism of action.

**Table 4:** Pharmacological causes of hypophosphatemia (prepared by the authors).

Mechanism	Drug class
Extrarenal (intracellular shift, decreased absorption)	
Calcium supplements	Oral calcium carbonate
Aluminum-containing antacids	Aluminum hydroxide
Sodium bicarbonate (high doses)	
Glucocorticoids	Prednisone, dexamethasone
Glucagon	
Oral phosphate supplements	Paradoxical effect (phosphate-induced FGF23 elevation)
Antiresorptives/bone-active agents	Bisphosphonates, denosumab, teriparatide
Renal (increased urinary phosphate loss)	
Antiretrovirals	Tenofovir disoproxil fumarate
Antineoplastics	Ifosfamide, imatinib, leflunomide, tyrosine kinase inhibitors
Diuretics	Acetazolamide, furosemide, thiazides
Intravenous iron	Ferric carboxymaltose

### 3.12 Limitations

Current review guidelines have several limitations that must be considered for optimal clinical application. One of the main barriers is the lack of standardization of methods, which generates variability in the results obtained and makes comparisons between different studies and laboratories difficult. This lack of methodological uniformity can compromise the reliability of diagnoses and therapeutic follow-up (113). In addition, instructions provided to patients are often not communicated effectively, which can lead to inadequate collection of biological samples and, therefore, to misinterpretations of clinical results. Another significant improvement lies in the use of popRI that may not be representative of the specific population under study (114). Tools such as Caliper, which are based on reference populations that do not necessarily reflect the demographic, socioeconomic, or ethnic characteristics of less developed groups or diverse ethnic backgrounds, can lead to discrepancies in the interpretation of biomarkers (29).

Diagnostic errors and delays continue to pose significant challenges in clinical practice. The initial misdiagnosis rate for TIO exceeds 95%, with an average diagnostic delay estimated at 4.8 years (5, 8). Similar issues arise with XLH, where diagnostic inaccuracies are common. Although the genetic nature of XLH can offer helpful diagnostic clues, the absence of standardized pediatric reference ranges for phosphate and alkaline phosphatase in clinical laboratories can result in both underdiagnosis and misclassification (115).

To improve the diagnostic accuracy and clinical management of patients with XLH and TIO, encouraging future research proposals that address current methodological and standardization deficiencies is necessary. One of the key priorities is the development of a standardized consensus that harmonizes the different detection methods used in clinical practice and research. This consensus should involve the collaboration of multiple health centers and experts in nephrology, rheumatology, endocrinology, and genetics to establish uniform protocols for sample collection, biochemical and genetic testing, and interpretation of results. In addition, multicenter studies are needed to validate these standardized methods in diverse populations, including those with different ethnic and socioeconomic backgrounds, to ensure the applicability and reliability of the results at the global level. In parallel, new biomarkers should be investigated that, when combined with existing biomarkers, can offer greater specificity and sensitivity in the early diagnosis of XLH and TIO. The integration of advanced technologies, such as high-throughput genomic sequencing and artificial intelligence for clinical data analysis, also represents a promising avenue for optimizing the detection and therapeutic monitoring of these disorders.

## 4 Conclusions

This biochemical assessment approach for managing hypophosphatemia associated with XLH and TIO addresses the challenges inherent to the interpretation of biomarkers in these phosphate metabolic disorders that the clinician faces. Given the remarkable heterogeneity in detection techniques and the biological variation observed for FGF23, serum phosphate, phosphaturia, PTH, vitamin D, and corrected calcium excretion rates, these recommendations propose a multidisciplinary approach to improve diagnostic accuracy and adequately monitor treatment, with the reference change value and pRI being more sensitive methods that could allow clinicians to detect changes in disease progression or treatment efficacy early in comparison with classical approaches (popRI or fixed cutoffs). The need to homogenize measurement methods to overcome the limitations of current tools and reduce discrepancies in results between different laboratories is worth highlighting. In addition, a critical and contextualized evaluation of biomarkers is recommended, considering patient individual and circumstantial variation, for a more precise and personalized interpretation of results. These recommendations can help guide clinicians toward more effective diagnosis and monitoring of XLH and TIO, highlighting the need to standardize biomarker reference intervals on the basis of the populations analyzed. A multidisciplinary approach involving close collaboration between laboratory medicine and clinical specialists is pivotal for diagnosing these patients.

## Data Availability

The original contributions presented in the study are included in the article/**Supplementary Material**. Further inquiries can be directed to the corresponding author.

## Supplementary material

Supplementary Figure 1 Flowchart of article selection.
