# Supplementary figures and images for: Biochemical evaluation of X-linked hypophosphatemia and tumor-induced osteomalacia: insights into diagnosis and management
Source: Front Endocrinol (Lausanne). 2025 Nov 25;16:1702656. doi: 10.3389/fendo.2025.1702656 (PMC12746488; doi:10.3389/fendo.2025.1702656)

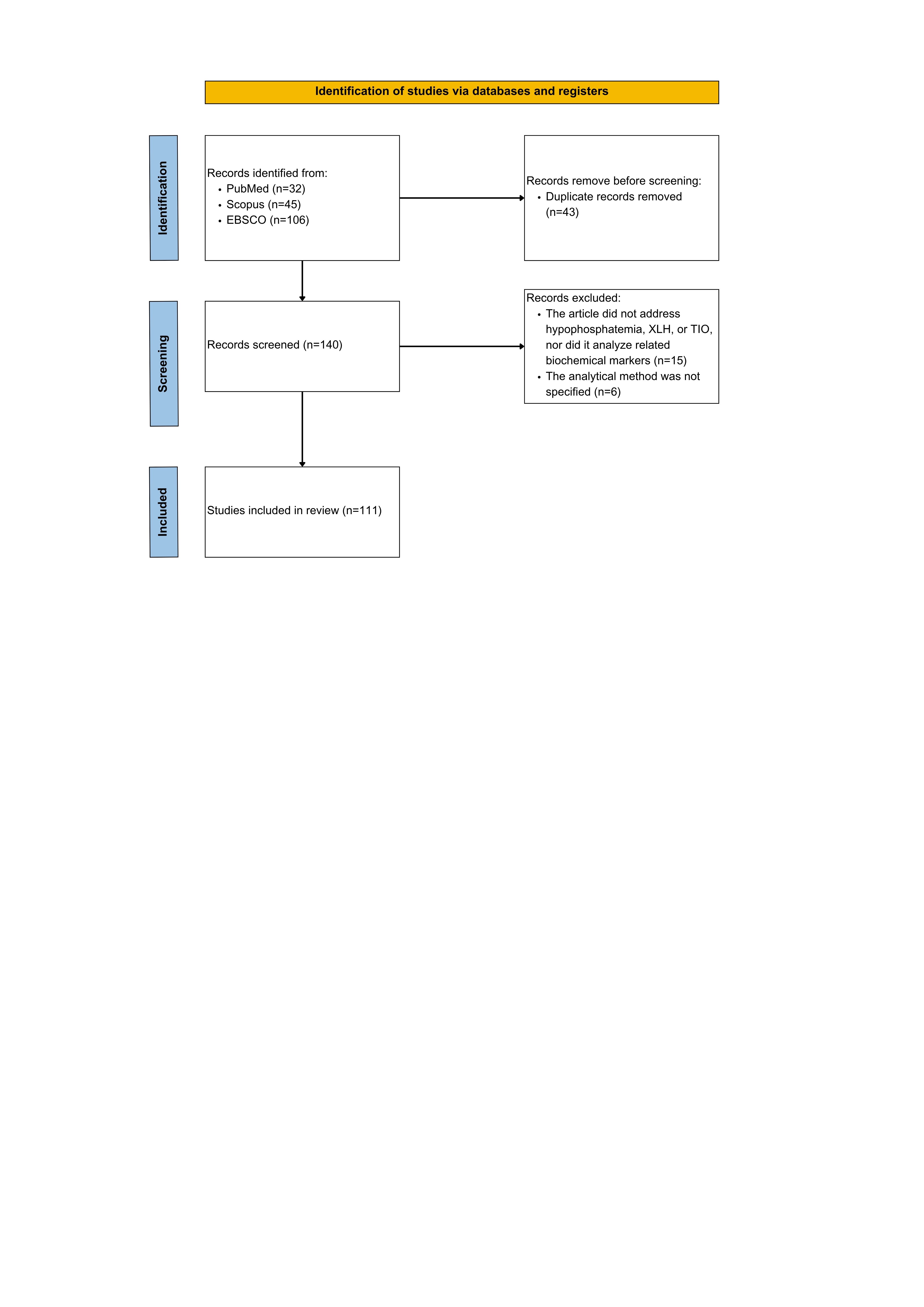

Supplement: Supplementary Figure 1 — Flowchart of article selection. [file Image1.jpg]
